# Melatonin Secretion Is Increased in Children with Severe Traumatic Brain Injury

**DOI:** 10.3390/ijms18051053

**Published:** 2017-05-13

**Authors:** Lucia Marseglia, Gabriella D’Angelo, Sara Manti, Immacolata Rulli, Vincenzo Salvo, Giuseppe Buonocore, Russel J. Reiter, Eloisa Gitto

**Affiliations:** 1 Department of Human Pathology in Adult and Developmental Age, University of Messina, 98125 Messina, Italy; gabdangelo@unime.it (G.A.); saramanti@hotmail.it (S.M.); rulli.imma@tiscali.it (I.R.); salvovincenzo@virgilio.it (V.S.); egitto@unime.it (E.G.); 2 Department of Molecular and Developmental Medicine, University of Siena, 53100 Siena, Italy; giuseppe.buonocore@unisi.it; 3 Department of Cellular and Structural Biology, University of Texas Health Science Center at San Antonio, San Antonio, 78229 TX, USA; reiter@uthscsa.edu

**Keywords:** traumatic brain injury, melatonin, pediatric intensive care, oxidative stress, antioxidant

## Abstract

Background: Traumatic brain injury (TBI) is a leading cause of death and disability in children. Oxidative stress plays a significant role in brain damage and melatonin exhibits both direct and indirect antioxidant effects. The primary aim of the present study was to evaluate serum melatonin levels in children with severe TBI in comparison to critically ill children admitted to the Pediatric Intensive Care Unit for conditions other than TBI. Methods: Twenty-four children were evaluated, equally divided into severe TBI and no-TBI. Blood samples for serum melatonin analysis were collected at 22:00, 01:00, 03:00, 05:00, 08:00, and 12:00. Results: Mean serum melatonin peaks in children of the TBI group were higher compared to the values of no-TBI critically ill children (495 ± 102 vs. 294 ± 119 pg/mL, *p* = 0.0002). Furthermore, the difference was even more significant in comparison to values reported in literature for healthy age-matched children (495 ± 102 vs. 197 ± 71 pg/mL, *p* < 0.0001). Conclusion: This study has shown that endogenous serum melatonin levels dramatically increase in children after severe TBI. This elevation is likely to represent a response to oxidative stress and/or inflammation due to severe head injury.

## 1. Introduction

Severe traumatic brain injury (TBI) is a leading cause of morbidity and mortality in children and represents a common cause of admission to Pediatric Intensive Care Units (PICUs). Disturbed sleep is a frequent problem both in ICU patients and in people with significant TBI, and has been mainly linked to abnormal melatonin production [1,2]. Melatonin is an endogenous indolamine, involved in the circadian regulation of the daily sleep–wake cycle, produced by the pineal gland at night under conditions of darkness in both diurnal and nocturnal species. Its rhythm of secretion is generated by an endogenous circadian master clock in the suprachiasmatic nucleus (SCN) of the hypothalamus, which is entrained by the light/dark cycle over a 24 h period [3]. Hence, melatonin secretion is normally low during daytime, increases soon after onset of darkness, and peaks in the middle of the night to gradually fall during the second half of the night. ICU and PICU [4] are noisier than usual hospital settings and constantly lit, therefore rhythmic signals from the environment are abolished. Studies performed in adult populations showed severely depressed melatonin secretion in critical care patients undergoing mechanical ventilation [5,6], and with severe sepsis [7], both in terms of nocturnal peaks and basal daytime serum levels [1,8]. Rajaratnam et al. recently showed that individuals with moderate to severe TBI had lower evening and overnight melatonin production than healthy individuals, suggesting that TBI may disrupt the melatonin synthesis pathway, leading to altered sleep–wake cycles [2,9]. Melatonin deficiency has been demonstrated to have deleterious effects in preclinical models of TBI [10], while exogenous administration has been shown to confer neuroprotective effects in animal models of TBI, reducing the degree of histologic damage and cerebral edema, as well as improving neurobehavioral outcomes [11,12]. Neural cell loss after TBI is triggered by a number of complex biochemical processes, including parenchymal inflammation, free radical (FR) production, increased intracellular calcium, lipid peroxidation and nitric oxide production [13,14]. Melatonin is a highly efficient antioxidant [15] and neuroprotective effects are principally related to its free radical scavenger activity in the central nervous system (CNS) [16].

In a previous study [17], in contrast with data obtained from adult ICU patients [1,5,6,7], we observed high melatonin peak levels in critically ill, sedated and mechanically ventilated, pediatric patients. We considered this finding as a physiologic counteracting response to oxidative stress (OS) connected to serious diseases. Among the study population, we noted the highest serum melatonin peaks in four children with TBI, but the sample size was too small to draw conclusions. Thus, the primary aim of the present study was to evaluate serum melatonin levels in critically ill children with TBI in comparison to critical children admitted to the PICU for conditions other than TBI, to deepen understanding on the link between severe TBI and melatonin secretion in children. The second aim was to study the circadian rhythm of serum melatonin levels and to test the effect of light on the rhythm in children with severe TBI.

## 2. Results

Although there was no significant gender difference in the total study population (13 males and 11 females), males predominated in the TBI patient group (*n* = 9, 75%). Motor vehicle accidents caused head injury in the majority of cases (*n* = 6, 50%), followed by pedestrian and motor cycle accidents accounting for 34% of cases, sports related head injury, and fall from building in one case each, overall accounting for 16% of TBI patients.

**Figure 1:**
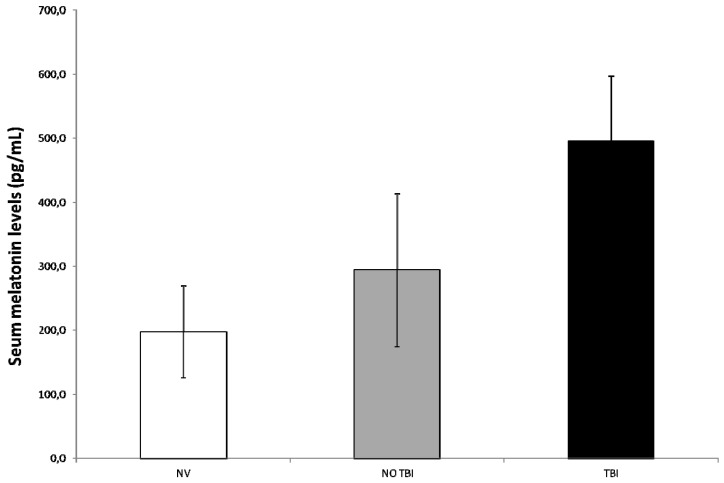
Mean serum melatonin peaks in children of the traumatic brain injury (TBI) group were significantly higher compared to the values of no-TBI critically ill children and to values reported in literature for healthy age-matched children. Melatonin levels were higher also in the no-TBI group than normal published values.

Mean serum melatonin peaks in children of the TBI group were higher compared to the values of no-TBI critically ill children (495 ± 102 vs. 294 ± 119 pg/mL, *p* = 0.0002) (Figure 1). Furthermore, as expected, the difference was even more significant in comparison to values reported in literature for healthy age-matched children [18] (495 ± 102 vs. 197 ± 71 pg/mL, *p* < 0.0001). Confirming previous results [17], melatonin levels were higher also in the no-TBI group than normal published values [18] (294 ± 119 vs. 197 ± 71 pg/mL, *p* = 0.025) (Figure 1).

**Figure 2:**
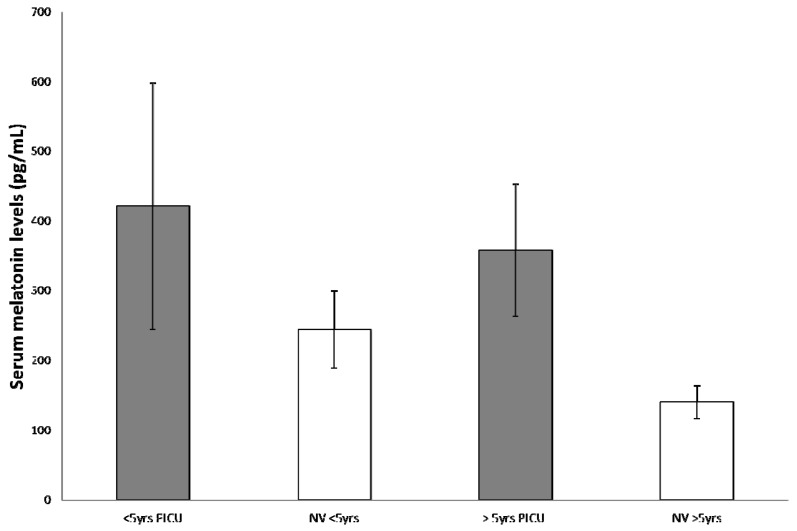
Independently of the diagnosis of Pediatric Intensive Care Unit (PICU) admission (TBI vs. no-TBI), the increase in melatonin secretion was significantly higher in children older than five years, in comparison to normal values (NV) reported in healthy age-matched children. These data confirm the peculiar role of critical conditions in the increase of melatonin secretion in children.

**Figure 3:**
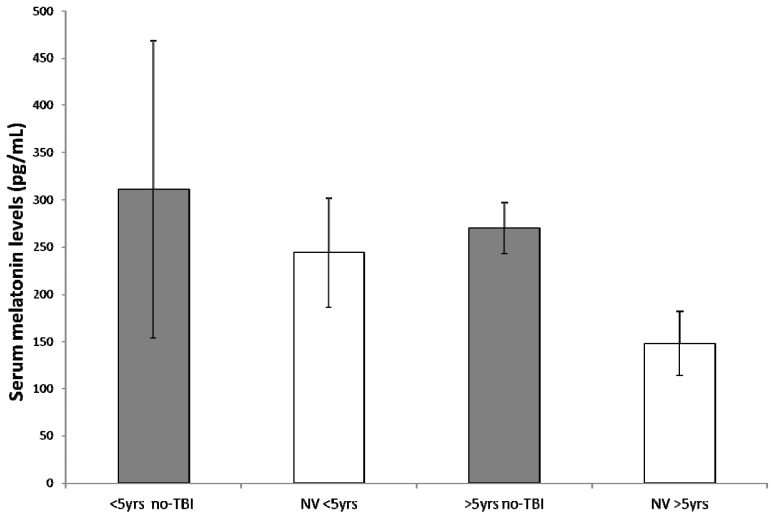
Considering age and principal diagnosis traumatic brain injury (TBI) vs. no-traumatic brain injury (no-TBI)), a non-statistically significant difference from normal values for children younger than five years in the no-TBI group was found. In children older than five years of the no-TBI group, melatonin levels were significantly higher compared with normal values reported in literature.

**Figure 4:**
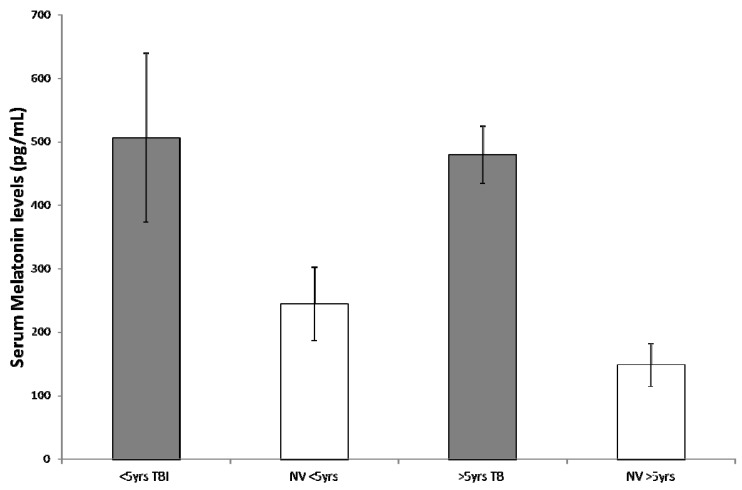
Both in children younger and older than five years within the traumatic brain injury (TBI) group, mean serum melatonin levels were significantly higher than normal literature values (NV), highlighting the role of head injury on the endogenous melatonin secretion.

To correlate patient’s age and melatonin secretion, we stratified the study population into 2 levels of age (<5 years and >5 years). In the younger group, independently of the diagnosis of PICU admission, we found a mean of 421 ± 177 vs. 244 ± 55 pg/mL reported in healthy age-matched children (*p* = 0.0014), whereas in the older group, mean melatonin value was 358 ± 95 vs. 140 ± 24 pg/mL in healthy subjects (*p* < 0.0001) (Figure 2). Considering age and principal diagnosis (TBI vs. no-TBI), we observed a non-statistically significant difference from normal values for children <5 years in the no-TBI group (311 ± 157 vs. 244 ± 58 pg/mL, *p* = 0.30); while in older children (>5 years) of the same group, we obtained a statistically significant difference with a mean of 270 ± 27 pg/mL compared with normal values reported in literature of 148 ± 34 pg/mL (*p* < 0.0005) (Figure 3). In the TBI group, mean serum melatonin levels were significantly higher than normal literature values both in younger and older children (506 ± 133 vs. 244 ± 58 pg/mL, *p* = 0.0004, and 479 ± 45 vs. 148 ± 34 pg/mL, *p* < 0.0001, respectively) (Figure 4).

Both TBI and no-TBI children enrolled in the dark group presented peak melatonin values which were significantly higher than those of the light group patients (mean value of melatonin in TBI dark group was 545 ± 101 vs. 425 ± 51 pg/mL in TBI light group, *p* = 0.036; in the no-TBI dark group it was 377 ± 105 vs. 211 ± 59 pg/mL in the no-TBI light group, *p* = 0.0073).

The serum melatonin rhythm was found to be altered in all but one patient in the no-TBI group dark exposed. Only four out of 24 patients presented a melatonin peak at 01:00 or 03:00: two were children with TBI in the dark group, and two were children light exposed of the no-TBI group.

No correlation between melatonin secretion and sedation drugs (fentanyl and midazolam) or inotropic therapy was observed. In this regard, it has already been reported that the biosyntheses of dopamine and melatonin are each critically related to the light-dark cycle [19]. It is known that dopamine produced primarily by the amacrine cells, mediates the effects of light causing the suppression of melatonin biosynthesis by the photoreceptor cells [20]. However, a reciprocal inhibitory relationship exists between dopamine and melatonin in the retina of mammals, as dopamine inhibits melatonin synthesis through D2/D4 receptors located within the neural retina [21].

Similarly, the correlation between body weight and melatonin values resulted in no statistically significant differences; likewise, there was no significant correlation between Pediatric Glasgow Coma Scale (PGCS) and melatonin levels in the TBI group.

## 3. Discussion

The present study demonstrated, for the first time, that the secretion of melatonin in children admitted in PICU with severe TBI is significantly enhanced. In a previous investigation [17], endogenous melatonin levels in critically-ill children were found to be significantly higher than those of age-matched healthy controls, contrary to what occurs in adult critical care patients, where melatonin secretion has been found to be suppressed [1,5,6,7]. Similarly, the results of the present study highlight the striking difference in melatonin production between children and adult patients with TBI. Despite the role of altered melatonin production in sleep disorders in individuals with TBI being generally accepted, in literature, only few studies have evaluated melatonin secretion in these patients and only three measured melatonin levels in adult TBI patients during an ICU stay [22,23,24]. First, Paparrigopulos and colleagues [22] found reduced blood melatonin in eight patients admitted to ICU following severe head injury, compared to reported normal values in literature. Moreover, melatonin secretion was closely related to the patients’ Glasgow coma score (GCS), with absent circadian pattern of serum melatonin in subjects with low GCS (<7) and retained melatonin normal fluctuations in those with high GCS (>7), indicating a possible relationship between severity of head injury and loss of physiological fluctuation of melatonin levels. In contrast with their findings, our results showed that in pediatric patients with TBI, melatonin was not correlated with PGCS, although the study included only children with severe TBI (PGCS ≤ 8; mean 5.8 ± 1.4) and the impact of moderate or mild brain injury could not be evaluated. Independently from PCGS, in our patients, the serum melatonin rhythm was found to be altered in all subjects of the TBI group, and in 11/12 patients of the no-TBI group, confirming that TBI and ICU are associated with grossly disturbed melatonin physiologic patterns, possibly leading to an altered sleep–wake cycle. Our data are partially in accord with results from Seifman et al. [23], who demonstrated, in the second study performed on critical care patients with TBI, derangement of the circadian rhythm of melatonin production in patients who had severe head injury, although they reported decreased melatonin concentrations in their adult subjects, in contrast to the increased levels that we found in children. The mechanism behind the disturbed melatonin secretion rhythm in ICU patients is not completely known, although environmental factors, primarily including light stimulus, are clearly involved. Light is the dominant factor that inhibits melatonin biosynthesis in the pineal gland [25]. We found that both TBI and no-TBI children enrolled in the light group presented peak melatonin values which were significantly lower than those of the dark group patients. Moreover, we found the highest mean melatonin peak in patients dark exposed from the TBI group. These data suggested a significant rise in the indolamine following severe head injury and in conditions of darkness, and an only partial inhibition by light on melatonin secretion.

In the third study [24], melatonin levels were measured both in serum and cerebrospinal fluid (CSF) of adult subjects admitted to ICU for TBI, and the relationship with isoprostane, a marker of OS, was evaluated. Moreover, they compared melatonin levels in CSF of subjects with TBI to those of patients undergoing elective neurosurgical procedures. Authors showed that CSF, but not serum melatonin levels, were markedly elevated only in subjects with TBI.

It has been furthermore demonstrated that melatonin levels in the third ventricle are several orders of magnitude higher than those in peripheral blood, as the pineal gland can directly release melatonin into the third ventricle via its recess, and the indolamine easily reaches other cerebral structures by active transportation, diffusion via ependymal cells and Virchow-Robin perivascular spaces [26]. The high levels of melatonin in CSF could provide antioxidative and anti-inflammatory protection for brain tissues. Therefore, the reported elevation of melatonin CSF, directly correlated with the heightened isoprostane levels, represented likely a response to OS [24]. The injured brain is highly susceptible to FR oxidative damage, for several reasons: (a) high levels of polyunsaturated fatty acids present in the myelin, a rich source for lipid peroxidation reactions [27]; (b) elevated rate of metabolic activity with marked reactive oxygen metabolite generation [28]; (c) high levels of transition metals, such as iron, which catalyze the production of reactive radicals [29]; (d) increased blood–brain barrier permeability [30]; and (e) vasogenic edema [31] and post traumatic microvascular damage [32]. Research has identified, among the sequelae of TBI, an increased production of FRs via a number of pathologic cascades that mainly include a cerebral inflammatory response with release of pro-inflammatory cytokines, such as interleukin-1b (IL-1b), IL-6, and IL-18, apoptosis induced by mitochondrial dysfunction, disruption of Ca^2^ homeostasis, excitotoxicity, and enhanced reactive oxygen species (ROS) production. It has been widely accepted that mitochondria represent the main source of cellular ROS, derived from the electron transport chain under several stress conditions, including TBI [33]. It has recently been demonstrated that TBIs cause structural and functional damage in mitochondria, leading to overproduction of ROS generated by the mitochondria, resulting in mitophagy, and finally in cell death [34].

The observed increase in melatonin concentrations, both in the CSF of adult patients with head injury [24] and serum of our pediatric population with severe TBI, may be rationalized in view of the deleterious OS resulting from TBI that would induce the overproduction of endogenous melatonin, as an antioxidant and neuroprotective agent. Melatonin, due to its pleiotropic properties, has been demonstrated to provide protection in brain injury models by stabilizing endothelial permeability, reducing OS and neuronal death following injury [11,12]. Besides its well known antioxidant actions [35,36], it has been recently demonstrated that melatonin represses TBI-induced inflammation by removing damaged mitochondria through the activation of mitophagy [37]. The clearance of damaged or malfunctioning mitochondria is essential for maintaining cellular homeostasis, and melatonin-mediated mitophagy is a critical regulatory mechanism for preventing hyperinflammation after TBI [37]. The results of the present study confirm that children with critical conditions respond to OS related to their serious conditions with overstimulation of pineal gland and higher endogenous melatonin levels. This phenomenon is particularly enhanced in the case of TBI, probably in view of melatonin’s effects as a neuroprotective agent. Unfortunately, in our study population, the evaluation of CSF melatonin values has not been performed. This represents a limitation of the study, that could have supported the hypothesis that the higher melatonin levels observed in children with TBI, in comparison to both healthy and critically-ill children without TBI, represent an attempt to counteract the deleterious consequences of oxidative damage following the brain injury. Moreover, our data suggest that in critical conditions the child’s pineal gland is over-stimulated and it could be hypothesized that this response is lost in parallel with the overall decline in melatonin levels described later in life, thus explaining the lower levels of melatonin observed in critically-ill adult patients in ICU.

## 4. Methods

### 4.1. Patients

This prospective study was conducted in the PICU at the University Hospital of Messina. Inclusion criteria were the following: age >1 month old, patients admitted to PICU for severe TBI, intubated and ventilated with an estimated need for mechanical ventilation of more than 2 days. At PICU admission, the Pediatric Glasgow Coma Scale (PGCS) was used to assess consciousness and classify the severity of head injuries, with scores for severe TBI (PGCS ≤ 8), moderate TBI (PGCS 9–12) and mild TBI (PGCS 13–15) [38]. Only patients with PGCS ≤ 8 were considered eligible for the present study.

Exclusion criteria were children’s legal guardian refusal to participate in the study. This study was approved by the ethics committee of the University Hospital of Messina (797/2012; June 2012) and written informed consent was obtained from the patients’ legal guardians.

In addition to the 4 patients with TBI already evaluated in the previous study [17], another 8 patients were enrolled (TBI group) and were compared to 12 critically ill children admitted with other diagnoses (no-TBI group) previously enrolled in the pilot study [17].

#### 4.1.1. TBI Group

In the TBI group, mean age was 5.5 years ± 2.1. Nine were males and 3 females. All children presented accidental head injury related to: motor vehicle accidents in 6 patients; bicycle and motor cycle related accidents in 2 and 1 patient, respectively; sports related head injury, pedestrian injury, and fall in one case each. The mean ± standard deviation (SD) for the PGCS was 5.8 ± 1.4.

All patients were mechanically ventilated and sedated with midazolam (2.7 ± 1.3 μg/kg/min) and fentanyl (3.8 ± 1.9 μg/kg/h). The attending intensivist was responsible for judging the adequacy of sedation level and the management of infusion rates, assessing the COMFORT score [39]. Eight patients required continuous intravenous inotropic support with dopamine (8.7 ± 2.5 μg/kg/min). None received other drugs known to interfere with melatonin metabolism such as β blocking agents, norepinephrine, corticosteroids or clonidine. None had liver or renal failure at the time of the study.

#### 4.1.2. No-TBI Group

In the no-TBI group, the mean age was 5.3 years ± 3.2. Four were males and 8 females. Principal diagnoses were the following: respiratory failure in 8 patients, pneumonia in 2, polytrauma and hemolytic uremic syndrome in one case each. All patients were mechanically ventilated and sedated with midazolam (1.8 ± 1.1 μg/kg/min) and fentanyl (2.1 ± 1.2 μg/kg/h). Five patients required continuous intravenous inotropic support with dopamine (6.8 ± 3.2 μg/kg/min). None received other drugs known to interfere with melatonin metabolism. None had liver or renal failure at the time of the study.

### 4.2. Experimental Design

At enrollment time, patients were randomly assigned to a dark or to a light group to evaluate the effects of light on serum melatonin concentrations. In the former group, all lights on the ward were turned off for subject patients at 23:00 h to complete darkness (<1 lux) until 07:00 h. In the latter group, artificial white light (500–800 lux), typical of the illumination of hospital rooms and similar to domestic lighting in Italy, remained on during the night.

The study started at 22:00 and lasted 14 hours. Blood samples for serum melatonin analysis were collected at 22:00, 01:00, 03:00, 05:00, 08:00, and 12:00 from central venous catheters placed in the femoral vein before the beginning of the study. Extreme attention was paid to maintaining darkness at night when the blood samples were taken via central venous catheters in patients included in the dark group; a dim light (<30 lux) was used for the collection of samples during the dark span. Samples were collected in plastic tubes without anticoagulant agents. Serum samples were immediately separated by centrifugation and stored at −20 °C until assayed.

### 4.3. Melatonin Assay

Melatonin was measured by an Enzyme-Linked Immunosorbent Assay (ELISA) kit (DRG Melatonin ELISA–EIA-1431, DRG International Inc., Mountainside, NJ, USA); sensitivity 1.60 pg/mL, intra-assay CV 3.0–11.4% between 8.8 and 151.7 pg/mL, inter-assay CV 6.4–19.3% between 5.6 and 134.3 pg/mL). Samples were not randomized to ELISA plates and, to try to prevent the microplate edge effects and decrease the overall evaporation, particular attention was paid to the amount of time fluids were stored in the well microplates.

### 4.4. Statistical Analysis

Statistical analysis of serum melatonin levels between both TBI and no-TBI groups, and light and dark groups was performed using Wilcoxon’s signed rank test. Spearman’s correlation coefficient was used to analyze the relationship between serum melatonin peak values, sedation and anthropological parameters, and in the TBI group with PGCS score. *p*-Values < 0.05 were considered statistically significant.

## 5. Conclusions

This study has shown that endogenous serum melatonin levels dramatically increase in children after severe TBI, in comparison to both normal reported values for age and critically ill children admitted in PICU for conditions other than head trauma. This elevation is likely to represent a response to OS and/or inflammation due to severe head injury. Further research, also involving the evaluation of melatonin in the CSF of brain injured children, is necessary to confirm the significance of our findings and the potential utility of melatonin administration as a neuroprotective agent in patients having sustained severe TBI.

## Abbreviations

CNS	Central nervous system
CSF	Cerebrospinal fluid
FR	Free radicals
GCS	Glasgow Coma Scale
ICU	Intensive Care Unit
IL	Interleukin
OS	Oxidative stress
PGCS	Pediatric Glasgow Coma Scale
PICU	Pediatric Intensive Care Unit
ROS	Reactive oxygen species
TBI	Traumatic brain injury
